# Di­aqua­bis­(pyridine-2-carboxyl­ato-κ^2^ *N*,*O*)zinc di­methyl­formamide hemisolvate

**DOI:** 10.1107/S1600536813018941

**Published:** 2013-07-13

**Authors:** Lilia Croitor, Diana Chisca, Eduard B. Coropceanu, Marina S. Fonari

**Affiliations:** a Institute of Applied Physics, Academy of Sciences of R. Moldova, Academy str. 5, MD2028 Chisinau, Republic of Moldova; b Institute of Chemistry, Academy of Sciences of R. Moldova, Academy str. 3, MD2028 Chisinau, Republic of Moldova

## Abstract

In the title compound, [Zn(C_6_H_4_NO_2_)_2_(H_2_O)_2_]·0.5C_3_H_7_NO, the Zn^II^ ion is coordinated in a distorted octa­hedral N_2_O_4_ environment by two *N*,*O*-chelating pyridine-2-carboxyl­ate ligands and two *cis* water mol­ecules. The chelating pyridine-2-carboxyl­ate ligands create two five-membered Zn/N/C/C/O rings, which form a dihedral angle of 86.4 (2)°. In the crystal, O—H⋯O hydrogen bonds link the complex mol­ecules into a two-dimensional network parallel to (100). The di­methyl­formamide solvent mol­ecule is disordered about a twofold rotation axis.

## Related literature

For background to polydentate ligands, see: Udvardy *et al.* (2013 ▶); Groni *et al.* (2008 ▶); Golenya *et al.* (2011 ▶); Ma *et al.* (2009 ▶). For related structures, see: Chen & Hu (2011 ▶); Li *et al.* (2008 ▶); Lumme *et al.* (1969 ▶); Takenaka *et al.* (1970 ▶); Uggla *et al.* (1969 ▶). For hydrogen-bond graph-set motifs, see: Bernstein *et al.* (1995 ▶).
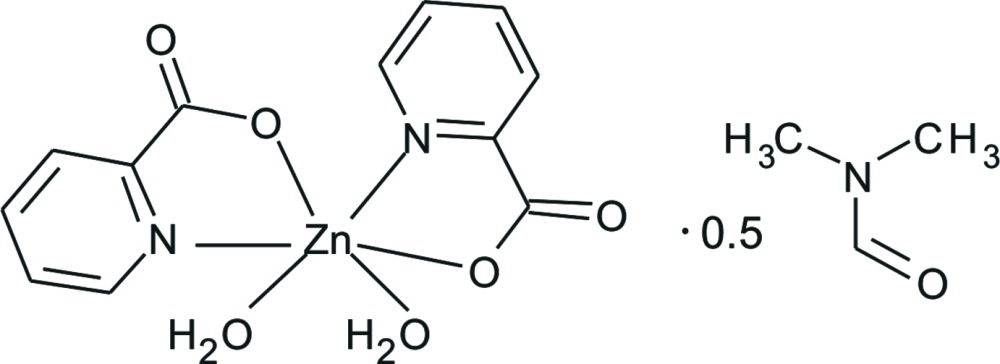


## Experimental

### 

#### Crystal data

[Zn(C_6_H_4_NO_2_)_2_(H_2_O)_2_]·0.5C_3_H_7_NO
*M* *_r_* = 382.16
Monoclinic, 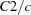

*a* = 25.777 (3) Å
*b* = 8.6754 (4) Å
*c* = 16.7916 (17) Å
β = 125.228 (15)°
*V* = 3067.4 (5) Å^3^
*Z* = 8
Mo *K*α radiation
μ = 1.64 mm^−1^
*T* = 293 K
0.18 × 0.12 × 0.02 mm

#### Data collection

Agilent Xcalibur Eos diffractometer
Absorption correction: multi-scan (*CrysAlis PRO*; Agilent, 2011 ▶) *T*_min_ = 0.906, *T*_max_ = 1.000
4865 measured reflections
2844 independent reflections
1772 reflections with *I* > 2σ(*I*)
*R*_int_ = 0.055

#### Refinement

*R*[*F* ^2^ > 2σ(*F* ^2^)] = 0.062
*wR*(*F* ^2^) = 0.114
*S* = 1.00
2844 reflections
244 parameters
162 restraints
H atoms treated by a mixture of independent and constrained refinement
Δρ_max_ = 0.47 e Å^−3^
Δρ_min_ = −0.44 e Å^−3^

### 

Data collection: *CrysAlis PRO* (Agilent, 2011 ▶); cell refinement: *CrysAlis PRO*; data reduction: *CrysAlis PRO*; program(s) used to solve structure: *SHELXS97* (Sheldrick, 2008 ▶); program(s) used to refine structure: *SHELXL97* (Sheldrick, 2008 ▶); molecular graphics: *SHELXTL* (Sheldrick, 2008 ▶); software used to prepare material for publication: *SHELXTL*.

## Supplementary Material

Crystal structure: contains datablock(s) I, New_Global_Publ_Block. DOI: 10.1107/S1600536813018941/lh5630sup1.cif

Structure factors: contains datablock(s) I. DOI: 10.1107/S1600536813018941/lh5630Isup2.hkl

Additional supplementary materials:  crystallographic information; 3D view; checkCIF report

## Figures and Tables

**Table 1:** Hydrogen-bond geometry (Å, °)

*D*—H⋯*A*	*D*—H	H⋯*A*	*D*⋯*A*	*D*—H⋯*A*
O1*W*—H1*W*1⋯O4^i^	0.86 (2)	1.86 (2)	2.715 (6)	174 (5)
O1*W*—H2*W*1⋯O2^ii^	0.86 (2)	1.89 (2)	2.723 (5)	163 (5)
O2*W*—H1*W*2⋯O2^iii^	0.86 (2)	1.98 (3)	2.768 (5)	152 (4)
O2*W*—H2*W*2⋯O3^i^	0.87 (2)	1.85 (2)	2.704 (5)	170 (4)

Symmetry codes: (i) 
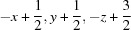
; (ii) 
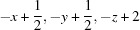
; (iii) 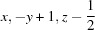.

**Fig. 1.**
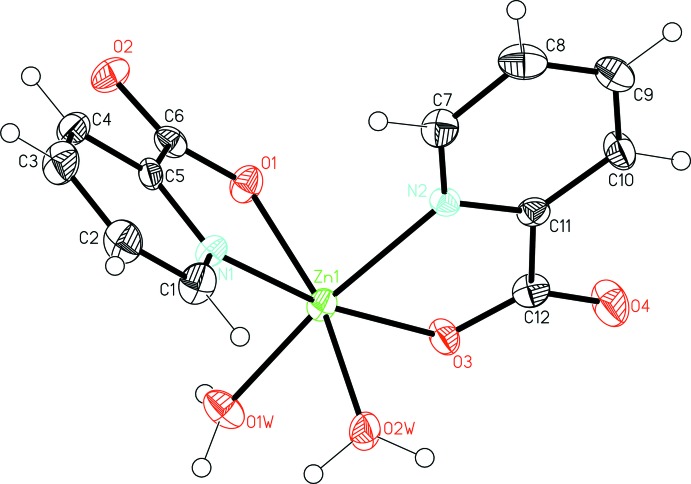
The molecular structure of [Zn(C6H4NO2)2(H2O)2]. Displacement ellipsoids are shown at the 30% probability level. H atoms are presented as small spheres of arbitrary radius.

**Fig. 2.**
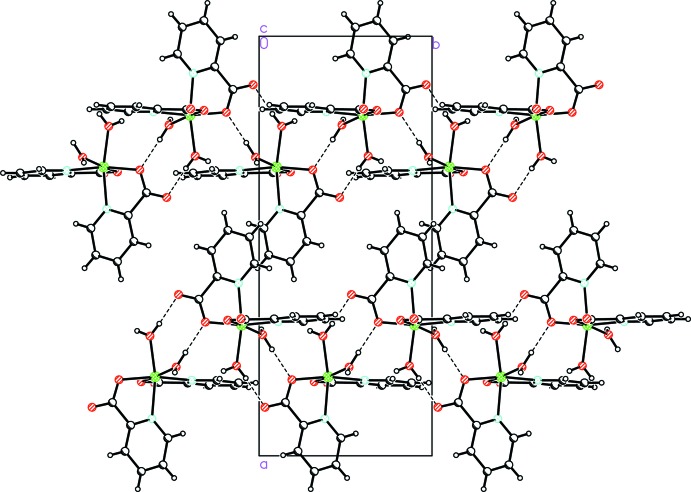
View along the crystallographic c axis. Solvent molecules are not shown. Hydrogen bonds are shown as dashed lines.

**Fig. 3.**
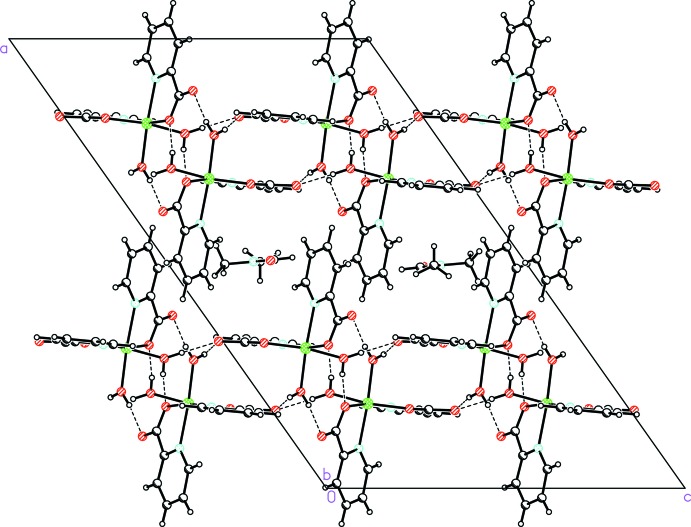
Part of the crystal structure showing incorporation of DMF molecules in the crystal. The view is along the crystallographic b axis. Hydrogen bonds are shown as dashed lines.

## supplementary crystallographic information

### Comment

Polydentate ligands such as pyridine-2-carboxylic acid (Hpic=picolinic acid) play important role in coordination chemistry and homogeneous catalysis. The combination of pyridyl and carboxyl groups in a single *bridge*-type ligand results in highly interconnected networks and gives limitless possibilities for increased network stability (Udvardy *et al.*, 2013; Groni *et al.*, 2008; Golenya *et al.* 2011). The COO^-^ group and the nitrogen atom in Hpic have strong coordination abilities and multiple coordination modes (Ma *et al.*, 2009). Herein, we report the crystal structure of the title compound.

The molecular structure of the title complex is shown in Fig. 1. The Zn^II^ ion is coordinated in a distorted octahedral N_2_O_4_ environment by two pyridine N-atoms, two carboxylate O atoms and two O atoms of two *cis*-coordinated water molecules. Each of two coordinated pic residues results in the formation of a five-membered chelate ring. The dihedral angle between two chelate rings is 86.4 (2)°. In some similar pyridine-2-carboxylato Zn(II) complexes (Chen & Hu, 2011; Li *et al.*, 2008; Lumme *et al.*, 1969; Takenaka *et al.*, 1970; Uggla *et al.*, 1969) the two pic ligands lie in the equatorial plane, and the water molecules are in a *trans-*arrangement.

The crystal packing is directed by hydrogen bond interactions with the participation of water H-donor atoms and carboxylic group O atoms acting as acceptors. Complex molecules are combined into ladder-like tapes *via* alternation of two similar *R*_2_^2^(8) (Bernstein *et al.*, 1995) graph-set motifs (Table 1, Figs. 2 and 3). The overall hydrogen bond motif is a two-dimensional layer parallel to (100). The title compound is isotypic with [Mn(C_6_H_4_NO_2_)_2_(H_2_O)_2_]^.^0.5C_2_H_3_N (Groni *et al.*, 2008), where the dimethylformamide solvent in the title compound is substituted by the acetonitrile.

### Experimental

To Zn(BF_4_)_2_(240 mg, 1 mmol) dissolved in 15 ml of water was added Hpic(246 mg, 2 mmol) dissolved in 15 ml of mixture methanol/dimethylformamide 1:1 (*v*/*v*). The reaction mixture was refluxed for ~ 15 min, and upon cooling to room temperature prism-shape colorless crystals precipitated (yield: 52%).

### Refinement

The C-bound hydrogen atoms were placed in calculated positions with C—H = 0.93Å and were treated using a riding-model approximation with *U*_iso_(H)=1.2*U*_eq_(C) or C—H = 0.96Å and *U*_iso_(H)=1.5*U*_eq_(C) for methyl H atoms. Water O—H hydrogen atoms were located from a difference Fourier map at intermediate stage of the refinement and the O—H and H···H distances were restrained to be 0.86 (1) and 1.46 (1) Å. These hydrogen atoms were refined with isotropic displacement parameter *U*_iso_(H)=1.5*U*_eq_(O). The dimethylformamide molecule is disordered about crystallographic twofold rotation axis.

### Crystal data

**Table:**

[Zn(C_6_H_4_NO_2_)_2_(H_2_O)_2_]·0.5C_3_H_7_NO	*F*(000) = 1568
*M**_r_* = 382.16	*D*_x_ = 1.655 Mg m^−^^3^
Monoclinic, *C*2/*c*	Mo *K*α radiation, λ = 0.71073 Å
Hall symbol: -C 2yc	Cell parameters from 957 reflections
*a* = 25.777 (3) Å	θ = 3.0–28.9°
*b* = 8.6754 (4) Å	µ = 1.64 mm^−^^1^
*c* = 16.7916 (17) Å	*T* = 293 K
β = 125.228 (15)°	Prism, colourless
*V* = 3067.4 (5) Å^3^	0.18 × 0.12 × 0.02 mm
*Z* = 8	

### Data collection

**Table:**

Agilent Xcalibur Eos diffractometer	2844 independent reflections
Radiation source: Enhance (Mo) X-ray Source	1772 reflections with *I* > 2σ(*I*)
Graphite monochromator	*R*_int_ = 0.055
Detector resolution: 15.9914 pixels mm^-1^	θ_max_ = 25.5°, θ_min_ = 3.0°
ω scans	*h* = −31→25
Absorption correction: multi-scan (*CrysAlis PRO*; Agilent, 2011)	*k* = −10→5
*T*_min_ = 0.906, *T*_max_ = 1.000	*l* = −9→20
4865 measured reflections	

### Refinement

**Table:**

Refinement on *F*^2^	Primary atom site location: structure-invariant direct methods
Least-squares matrix: full	Secondary atom site location: difference Fourier map
*R*[*F*^2^ > 2σ(*F*^2^)] = 0.062	Hydrogen site location: inferred from neighbouring sites
*wR*(*F*^2^) = 0.114	H atoms treated by a mixture of independent and constrained refinement
*S* = 1.00	*w* = 1/[σ^2^(*F*_o_^2^) + (0.025*P*)^2^] where *P* = (*F*_o_^2^ + 2*F*_c_^2^)/3
2844 reflections	(Δ/σ)_max_ = 0.001
244 parameters	Δρ_max_ = 0.47 e Å^−^^3^
162 restraints	Δρ_min_ = −0.44 e Å^−^^3^

### Special details

**Table:**

Geometry. All e.s.d.'s (except the e.s.d. in the dihedral angle between two l.s. planes) are estimated using the full covariance matrix. The cell e.s.d.'s are taken into account individually in the estimation of e.s.d.'s in distances, angles and torsion angles; correlations between e.s.d.'s in cell parameters are only used when they are defined by crystal symmetry. An approximate (isotropic) treatment of cell e.s.d.'s is used for estimating e.s.d.'s involving l.s. planes.
Refinement. Refinement of *F*^2^ against ALL reflections. The weighted *R*-factor *wR* and goodness of fit *S* are based on *F*^2^, conventional *R*-factors *R* are based on *F*, with *F* set to zero for negative *F*^2^. The threshold expression of *F*^2^ > σ(*F*^2^) is used only for calculating *R*-factors(gt) *etc*. and is not relevant to the choice of reflections for refinement. *R*-factors based on *F*^2^ are statistically about twice as large as those based on *F*, and *R*- factors based on ALL data will be even larger.

### Fractional atomic coordinates and isotropic or equivalent isotropic displacement parameters (Å^2^)

**Table:**

	*x*	*y*	*z*	*U*_iso_*/*U*_eq_	Occ. (<1)
Zn1	0.18837 (3)	0.40000 (7)	0.78138 (4)	0.0278 (2)	
N1	0.18028 (19)	0.6106 (5)	0.8391 (3)	0.0273 (11)	
N2	0.08845 (19)	0.3806 (5)	0.6684 (3)	0.0253 (10)	
O1	0.17673 (17)	0.3193 (4)	0.8877 (2)	0.0338 (10)	
O2	0.17327 (17)	0.3959 (4)	1.0115 (2)	0.0367 (10)	
O3	0.18125 (17)	0.1902 (4)	0.7129 (3)	0.0335 (10)	
O4	0.11570 (19)	0.0343 (5)	0.5895 (3)	0.0555 (13)	
C1	0.1798 (3)	0.7558 (6)	0.8114 (4)	0.0396 (16)	
H1	0.1837	0.7722	0.7603	0.048*	
C2	0.1738 (3)	0.8813 (6)	0.8553 (4)	0.0433 (16)	
H2	0.1739	0.9808	0.8347	0.052*	
C3	0.1677 (3)	0.8573 (7)	0.9300 (4)	0.0441 (17)	
H3	0.1633	0.9405	0.9606	0.053*	
C4	0.1681 (2)	0.7106 (6)	0.9593 (4)	0.0336 (14)	
H4	0.1646	0.6931	1.0107	0.040*	
C5	0.1738 (2)	0.5873 (6)	0.9120 (3)	0.0256 (13)	
C6	0.1751 (2)	0.4195 (6)	0.9396 (4)	0.0279 (13)	
C7	0.0422 (3)	0.4729 (6)	0.6531 (4)	0.0364 (15)	
H7	0.0529	0.5598	0.6921	0.044*	
C8	−0.0215 (3)	0.4441 (7)	0.5811 (4)	0.0461 (17)	
H8	−0.0529	0.5086	0.5735	0.055*	
C9	−0.0372 (3)	0.3196 (7)	0.5217 (4)	0.0428 (16)	
H9	−0.0794	0.2990	0.4717	0.051*	
C10	0.0109 (3)	0.2248 (7)	0.5376 (4)	0.0376 (15)	
H10	0.0014	0.1393	0.4980	0.045*	
C11	0.0729 (3)	0.2570 (6)	0.6122 (4)	0.0268 (13)	
C12	0.1270 (3)	0.1516 (6)	0.6384 (4)	0.0326 (14)	
O1W	0.28549 (18)	0.3692 (5)	0.8823 (3)	0.0389 (10)	
H1W1	0.3148 (18)	0.425 (5)	0.888 (4)	0.05 (2)*	
H2W1	0.298 (2)	0.296 (4)	0.924 (3)	0.042 (19)*	
O2W	0.2112 (2)	0.5202 (5)	0.6968 (3)	0.0413 (11)	
H1W2	0.199 (2)	0.512 (5)	0.6369 (16)	0.031 (16)*	
H2W2	0.2454 (17)	0.575 (6)	0.732 (3)	0.05 (2)*	
O1X	0.9935 (9)	0.7274 (12)	0.7077 (8)	0.142 (8)	0.50
N1X	0.9961 (14)	0.9688 (8)	0.7567 (15)	0.065 (4)	0.50
C1X	0.9916 (9)	0.8645 (13)	0.6968 (9)	0.093 (8)	0.50
H1X	0.9865	0.8999	0.6404	0.112*	0.50
C2X	1.0023 (11)	0.9255 (17)	0.8428 (12)	0.090 (7)	0.50
H2XA	0.9611	0.9229	0.8307	0.135*	0.50
H2XB	1.0286	0.9990	0.8933	0.135*	0.50
H2XC	1.0214	0.8252	0.8629	0.135*	0.50
C3X	0.996 (3)	1.1310 (9)	0.739 (3)	0.194 (10)	0.50
H3XA	1.0385	1.1673	0.7715	0.292*	0.50
H3XB	0.9750	1.1854	0.7631	0.292*	0.50
H3XC	0.9731	1.1486	0.6700	0.292*	0.50

### Atomic displacement parameters (Å^2^)

**Table:**

	*U*^11^	*U*^22^	*U*^33^	*U*^12^	*U*^13^	*U*^23^
Zn1	0.0328 (4)	0.0260 (4)	0.0265 (4)	−0.0006 (3)	0.0182 (3)	−0.0013 (3)
N1	0.034 (3)	0.026 (2)	0.026 (2)	0.001 (2)	0.020 (2)	0.002 (2)
N2	0.024 (3)	0.024 (3)	0.028 (3)	0.001 (2)	0.015 (2)	0.000 (2)
O1	0.045 (3)	0.027 (2)	0.032 (2)	−0.002 (2)	0.024 (2)	−0.0019 (18)
O2	0.046 (3)	0.041 (2)	0.032 (2)	0.005 (2)	0.027 (2)	0.0093 (19)
O3	0.024 (2)	0.033 (2)	0.034 (2)	0.0005 (19)	0.011 (2)	−0.0076 (19)
O4	0.039 (3)	0.047 (3)	0.065 (3)	0.000 (2)	0.022 (3)	−0.030 (2)
C1	0.057 (5)	0.029 (3)	0.038 (4)	−0.006 (3)	0.030 (4)	0.004 (3)
C2	0.053 (4)	0.022 (3)	0.053 (4)	−0.008 (3)	0.030 (4)	−0.006 (3)
C3	0.053 (5)	0.032 (4)	0.049 (4)	0.003 (3)	0.030 (4)	−0.011 (3)
C4	0.039 (4)	0.037 (4)	0.031 (3)	−0.002 (3)	0.024 (3)	−0.003 (3)
C5	0.019 (3)	0.029 (3)	0.020 (3)	−0.001 (3)	0.006 (3)	−0.006 (3)
C6	0.021 (3)	0.030 (3)	0.027 (3)	0.000 (3)	0.010 (3)	0.005 (3)
C7	0.039 (4)	0.028 (3)	0.041 (4)	0.008 (3)	0.023 (3)	−0.001 (3)
C8	0.036 (4)	0.045 (4)	0.062 (5)	0.019 (3)	0.031 (4)	0.013 (3)
C9	0.026 (4)	0.050 (4)	0.046 (4)	0.001 (3)	0.017 (3)	0.001 (4)
C10	0.029 (4)	0.036 (4)	0.038 (4)	−0.005 (3)	0.013 (3)	−0.010 (3)
C11	0.032 (3)	0.024 (3)	0.031 (3)	−0.002 (3)	0.021 (3)	0.001 (3)
C12	0.037 (4)	0.025 (3)	0.040 (4)	0.005 (3)	0.025 (3)	0.003 (3)
O1W	0.030 (3)	0.032 (3)	0.041 (3)	−0.005 (2)	0.013 (2)	0.012 (2)
O2W	0.049 (3)	0.049 (3)	0.026 (2)	−0.020 (2)	0.022 (2)	−0.005 (2)
O1X	0.117 (11)	0.095 (9)	0.19 (2)	0.029 (11)	0.074 (17)	−0.078 (9)
N1X	0.123 (11)	0.043 (6)	0.074 (8)	0.023 (15)	0.082 (8)	0.005 (13)
C1X	0.072 (14)	0.13 (2)	0.071 (16)	0.034 (17)	0.040 (14)	−0.035 (15)
C2X	0.132 (18)	0.084 (15)	0.072 (14)	0.015 (12)	0.069 (14)	0.015 (10)
C3X	0.45 (3)	0.055 (9)	0.26 (2)	0.02 (5)	0.31 (3)	0.03 (3)

### Geometric parameters (Å, º)

**Table:**

Zn1—O1W	2.078 (4)	C7—H7	0.9300
Zn1—O1	2.094 (3)	C8—C9	1.363 (7)
Zn1—O2W	2.101 (4)	C8—H8	0.9300
Zn1—O3	2.104 (3)	C9—C10	1.379 (7)
Zn1—N1	2.134 (4)	C9—H9	0.9300
Zn1—N2	2.150 (4)	C10—C11	1.375 (7)
N1—C1	1.341 (6)	C10—H10	0.9300
N1—C5	1.343 (6)	C11—C12	1.506 (7)
N2—C11	1.329 (6)	O1W—H1W1	0.857 (18)
N2—C7	1.333 (6)	O1W—H2W1	0.857 (18)
O1—C6	1.249 (6)	O2W—H1W2	0.863 (18)
O2—C6	1.251 (6)	O2W—H2W2	0.866 (19)
O3—C12	1.271 (6)	O1X—C1X	1.1999
O4—C12	1.233 (6)	N1X—C1X	1.3069
C1—C2	1.373 (7)	N1X—C2X	1.4118
C1—H1	0.9300	N1X—C3X	1.4373
C2—C3	1.368 (7)	C1X—H1X	0.9300
C2—H2	0.9300	C2X—H2XA	0.9600
C3—C4	1.362 (7)	C2X—H2XB	0.9600
C3—H3	0.9300	C2X—H2XC	0.9600
C4—C5	1.390 (7)	C3X—H3XA	0.9600
C4—H4	0.9300	C3X—H3XB	0.9600
C5—C6	1.523 (7)	C3X—H3XC	0.9600
C7—C8	1.389 (7)		
			
O1W—Zn1—O1	87.68 (16)	N1—C5—C6	115.4 (4)
O1W—Zn1—O2W	86.53 (18)	C4—C5—C6	123.6 (5)
O1—Zn1—O2W	167.39 (15)	O1—C6—O2	126.5 (5)
O1W—Zn1—O3	91.07 (15)	O1—C6—C5	117.2 (5)
O1—Zn1—O3	99.57 (14)	O2—C6—C5	116.3 (5)
O2W—Zn1—O3	91.74 (16)	N2—C7—C8	122.5 (5)
O1W—Zn1—N1	97.31 (16)	N2—C7—H7	118.8
O1—Zn1—N1	78.44 (15)	C8—C7—H7	118.8
O2W—Zn1—N1	91.19 (17)	C9—C8—C7	118.9 (5)
O3—Zn1—N1	171.28 (15)	C9—C8—H8	120.6
O1W—Zn1—N2	167.46 (16)	C7—C8—H8	120.6
O1—Zn1—N2	92.17 (15)	C8—C9—C10	118.4 (6)
O2W—Zn1—N2	95.90 (16)	C8—C9—H9	120.8
O3—Zn1—N2	76.58 (15)	C10—C9—H9	120.8
N1—Zn1—N2	94.94 (16)	C11—C10—C9	119.9 (5)
C1—N1—C5	118.5 (5)	C11—C10—H10	120.1
C1—N1—Zn1	129.1 (4)	C9—C10—H10	120.1
C5—N1—Zn1	112.4 (3)	N2—C11—C10	121.8 (5)
C11—N2—C7	118.5 (5)	N2—C11—C12	115.7 (5)
C11—N2—Zn1	114.0 (4)	C10—C11—C12	122.5 (5)
C7—N2—Zn1	127.4 (4)	O4—C12—O3	125.2 (5)
C6—O1—Zn1	116.2 (3)	O4—C12—C11	118.9 (5)
C12—O3—Zn1	117.7 (3)	O3—C12—C11	115.9 (5)
N1—C1—C2	122.7 (5)	Zn1—O1W—H1W1	126 (3)
N1—C1—H1	118.7	Zn1—O1W—H2W1	118 (3)
C2—C1—H1	118.7	H1W1—O1W—H2W1	116 (3)
C3—C2—C1	118.7 (5)	Zn1—O2W—H1W2	133 (3)
C3—C2—H2	120.6	Zn1—O2W—H2W2	113 (3)
C1—C2—H2	120.6	H1W2—O2W—H2W2	113 (3)
C4—C3—C2	119.5 (5)	C1X—N1X—C2X	120.7
C4—C3—H3	120.3	C1X—N1X—C3X	122.1
C2—C3—H3	120.3	C2X—N1X—C3X	117.2
C3—C4—C5	119.7 (5)	O1X—C1X—N1X	126.3
C3—C4—H4	120.2	O1X—C1X—H1X	116.9
C5—C4—H4	120.2	N1X—C1X—H1X	116.9
N1—C5—C4	121.0 (5)		

### Hydrogen-bond geometry (Å, º)

**Table:**

*D*—H···*A*	*D*—H	H···*A*	*D*···*A*	*D*—H···*A*
O1*W*—H1*W*1···O4^i^	0.86 (2)	1.86 (2)	2.715 (6)	174 (5)
O1*W*—H2*W*1···O2^ii^	0.86 (2)	1.89 (2)	2.723 (5)	163 (5)
O2*W*—H1*W*2···O2^iii^	0.86 (2)	1.98 (3)	2.768 (5)	152 (4)
O2*W*—H2*W*2···O3^i^	0.87 (2)	1.85 (2)	2.704 (5)	170 (4)

Symmetry codes: (i) −*x*+1/2, *y*+1/2, −*z*+3/2; (ii) −*x*+1/2, −*y*+1/2, −*z*+2; (iii) *x*, −*y*+1, *z*−1/2.
